# Influence of Pregnancy History on the Vaginal Microbiome of Pregnant Women in their First Trimester

**DOI:** 10.1038/s41598-017-09857-z

**Published:** 2017-08-31

**Authors:** Dimitrios Nasioudis, Larry J. Forney, G. Maria Schneider, Karol Gliniewicz, Michael France, Allison Boester, Mio Sawai, Jessica Scholl, Steven S. Witkin

**Affiliations:** 1 Division of Immunology and Infectious Disease, Department of Obstetrics and Gynecology, Weill Cornell Medicine, New York, New York, USA; 2 Department of Biological Sciences and Institute for Bioinformatics and Evolutionary Studies, University of Idaho, Moscow, Idaho USA

## Abstract

Pregnancy permanently alters maternal anatomy, physiology and immunity. We evaluated if the vaginal microbiome differed between women with a first or subsequent conception. Relative abundance of bacteria in the vaginal microbiome in first trimester pregnant women, 52 with their first known conception, 26 with a prior spontaneous or induced abortion but no deliveries and 77 with at least one prior birth, was determined by classifying DNA sequences from the V1-V3 region of bacterial 16 S rRNA genes. *Lactobacillus crispatus* was the numerically most abundant bacterium in 76.4% of women with a first conception, 50.0% with only a prior spontaneous or scheduled abortion and 22.2% with a prior birth (p ≤ 0.01). *L. iners* was the most abundant bacterium in 3.8% of women with a first conception as compared to 19.2% (p = 0.03) and 20.8% (p = 0.03) in those with a prior abortion or birth, respectively. *Gardnerella* as the most abundant bacterial genus increased from 3.8% in women with a first conception to 15.4% and 14.3% in those with a prior abortion or birth, respectively (p > 0.05). *L. iners* dominance was also associated with a history of spontaneous abortion (p ≤ 0.02). The composition of the vaginal microbiome and its influence on pregnancy outcome varies with pregnancy history.

## Introduction

*Lactobacillus* species tend to dominate the vaginal microbiome to a greater extent during pregnancy than when women are not pregnant^1, 2^. Their increased abundance and stability may be due to a multitude of factors that favor their selection. These might include the absence of cyclic hormonal variations or menstruation, decreased sexual activity, dietary and hygiene changes and alterations in the composition of cervical-vaginal secretions. The increased availability of specific nutrients might also enrich for lactobacilli. For example, estrogen production following conception promotes glycogen production in vaginal epithelial cells^3^, and glycogen degradation by α-amylase is thought to provide high levels of maltose oligomers that are readily metabolized by lactobacilli^4^. Additional contributing factors could be the immunological and physiological changes that occur during the transition from the non-pregnant to the pregnant state.

The influence of the vaginal microbiome on pregnancy outcome remains unsettled. It has been reported that the vaginal microbiota differs^5^ or does not differ^6^ between women who deliver prematurely or at term. However, the influence of a prior conception on the composition of the vaginal microbiota during gestation has not previously been investigated. In the present study we evaluated whether the vaginal microbiota in first trimester pregnant women varied depending on whether or not this was their first conception or first delivery. This time period was studied because successful implantation of the embryo into the uterine wall and initiation of vascular changes leading to development of the placenta during the first trimester may be the stages most susceptible to pregnancy disruption^7^.

## Results

Characteristics of the study population – women with their first known conception, women with no deliveries who had either a prior pregnancy termination or a spontaneous abortion, women with a prior delivery - are provided in Table 1. Women with their first conception were younger (22.3 years) than women in the other groups (p ≤ 0.01). There were no differences between groups in body mass index, the percentage who conceived by *in vitro* fertilization, the occurrence of a preterm delivery, gestational age at delivery or the neonatal birthweight. There were also no differences in the racial distribution of subjects in each group (Table 1).

**Table 1:** Characteristics of the study population.

Characteristic	First	Prior	Spont.	Prior
	conception	termination	abortion	delivery
Number women evaluated	52	10	16	77
Mean age (SD in years	32.3 (3.8)	33.3 (4.7)	36.0 (5.5)	34.6 (3.7)
Mean body mass index (SD)	22.5 (2.6)	22.9 (2.2)	23.0 (3.1)	22.4 (3.1)
*In vitro* fertilization	6 (11.3%)	3 (30.0%)	5 (29.4%)	2 (2.6%)
Preterm delivery	2 (3.8%)	0	0	1 (1.3%)
Mean gestational age at birth (SD)	39.2 (1.5)	39.7 (0.6)	39.3 (1.1)	39.5 (1.0)
Mean neonatal birthweight (SD) g	3294 (513)	3306 (404)	3355 (534)	3287 (500)
Race				
White	52.9%	37.5%	46.7%	63.5%
Asian	13.7%	25.0%	13.3%	10.8%
Hispanic	5.9%	0	6.7%	2.7%
Black	2.0%	0	0	0
Mixed	25.5%	37.5%	33.3%	23.0%

Spont, spontaneous; SD; standard deviation.

### Characterization of bacterial communities in relation to pregnancy history

The association between a prior conception or birth and the relative abundance of various bacterial taxa in the vaginal microbiome is shown in Table 2. *L. crispatus* was the numerically most abundant bacterium in 76.4% of women with a first conception, in 50.0% of women with a prior spontaneous or induced abortion but no births and in 22.1% of women with a prior birth. These differences in *L. crispatus* abundance between women with their first conception and those with a prior birth (p = 0.0001) and between women with a prior abortion only and those with a prior birth (p = 0.0116) were significant. Conversely, *L. iners* was the numerically most abundant bacterium in only 3.8% of women with a first conception as opposed to 19.2% with a prior abortion (p = 0.0378) and 20.8% in women with a prior birth (p = 0.0032). Similarly, detection of *L. gasseri* as the most abundant vaginal bacterium occurred in 1.9% of women with a first conception *as* opposed to *7.7%* with a history of spontaneous abortions and 18.2% of women with a previous birth (p = 0.0043 vs. first conception). The presence of *Gardnerella* as the numerically most abundant bacterial genus also increased from 3.8% of women in the first conception group to 15.4% in the abortion only group and 14.3% in the prior birth group. However, these differences did not reach statistical significance (p > 0.05), possibly due to the small number of women in these groups. The relative abundance of the different bacterial taxa were similar between White and non-White subjects (Table 2). There were no differences in the relative abundance of the bacterial taxa between women with no deliveries who had either a spontaneous pregnancy loss or induced termination (data not shown). Similarly, there were no associations between the abundance of any bacterial taxa and time since spontaneous abortion or birth.

**Table 2:** The numerically most abundant bacterial taxa in the vaginal microbiota of pregnant women in the first trimester in relation to pregnancy history and race.

Bacterial taxa	First conception	Prior conception but no births	Prior birth	White	Non-white
	N = 52	N = 26	N = 77	N = 84	N = 67
*L. crispatus*	76.4%^a^	50.0%^b^	22.1%	46.4%	38.8%
*L. iners*	3.8%^c^	19.2%	20.8%	7.1%	10.4%
*L. gasseri*	1.9%	7.7%	18.2%^d^	10.7%	11.9%
*L. jensenii*	9.6%	7.7%	5.2%	4.8%	11.9%
Other lactobacilli	0%	0%	6.5%	3.6%	0
*Gardnerella*	3.8%^e^	15.4%	14.3%	8.3%	10.4%
*Streptococcus*	3.8%	0%	5.2%	1.2%	6.0%
Other genera	0.7%	0%	7.7%	3.6%	7.5%

The data are presented as the percentage of cases in which a specific taxa was numerically most abundant in each of the three categories of subjects.

^a^p = 0.0001 vs. prior delivery, 0.0217 vs. prior conception; ^b^p = 0.0116 vs. prior delivery; ^c^p = 0.0032 vs. prior delivery, 0.0378 vs. prior conception; ^d^p = 0.0043 vs. first conception; ^e^p = 0.0914 vs. prior conception, 0.0734 vs. prior delivery.

*L. crispatus, L. iners* or *L. jensenii* were each the numerically most abundant vaginal bacteria in one of the three women who experienced a preterm birth while the six women who experienced a spontaneous abortion had vaginal microbiotas in which *L. crispatus (*two women*), L. jensenii, Bifidobacterium, Gardnerella* or *Streptococcus* were most abundant. There were no statistically significant associations between the relative abundance of any bacterial taxa and maternal age, gestational age at birth, or neonatal gender.

A listing of all taxa in the vaginal microbiome of each subject in each of the three groups is presented in supplemental Tables 1–3.

**Table 3:** The numerically most abundant bacterial taxa in vaginal communities of 155 pregnant women during the first trimester in relation to history of spontaneous abortion.

Bacterial taxa	Number of prior abortions			
	None	One	Two	Three
	N = 116	N = 26	N = 11	N = 2
*L. crispatus*	48.3%	38.5%	27.3%	50%
*L. iners*	9.5%	26.9%^a^	45.4%^b^	0%
*L. gasseri*	9.5%	23.1%	0%	0%
*L. jensenii*	8.6%	0%	0%	50%
Other lactobacilli	4.3%	0%	0%	0%
*Gardnerella*	12.1%	0%	27.3%	0%
*Streptococcus*	5.2%	0%	0%	0%
Other genera	2.5%	11.5%	0%	0%

The data are presented as the percentage of cases in which a specific taxa was numerically most abundant in each of the abortion categories.

^a^p = 0.0240 vs. none; ^b^p = 0.0049 vs. none.

### Vaginal communities and history of spontaneous abortions

Among the women in both the prior conception only and birth groups, 39 (25.2%) had a history of spontaneous abortion. Twenty six women (16.8%) had one abortion, 11 (7.1%) had two abortions and two (1.3%) had three abortions. The association between spontaneous abortion history and bacterial abundance is detailed in Table 3. The only significant difference noted was with *L. iners*. This bacterium was numerically most abundant in 9.5% of the women who had never had a spontaneous abortion as compared to 26.9% with one abortion (p = 0.0240) and 45.4% with two abortions (p = 0.0049). In the two women with three spontaneous abortions *L. crispatus* or *L. jensenii* were most abundant in their vaginas. There was no association between a history of spontaneous abortion and maternal age or gestational age at the time of birth.

## Discussion

Here we provide evidence that a prior conception influences the relative composition of the vaginal microbiota during the first trimester of pregnancy. Women with a prior birth or even only an induced or spontaneous abortion had a vaginal microbiota in the index pregnancy that was qualitatively and quantitatively different from that present in women experiencing their first conception. Specifically, there was a marked decrease in the relative abundance of *L. crispatus* and a concomitant increase in the relative abundance of other *Lactobacillus* species as well as *Gardnerella* in women with a prior conception, regardless of whether or not this proceeded to a birth. It appears that alterations in the vaginal microenvironment in women with a prior conception or delivery favor the proliferation of *Gardnerella, L. iners* and *L. gasseri* at the expense of *L. crispatus*.

What are the implications of our observation that the vaginal microbiota differs depending on prior pregnancy history? First of all it suggests that future studies designed to address the influence of the vaginal microbiome on pregnancy outcome must invariably take into account the pregnancy history of the women being evaluated. In addition, the generally accepted increase in *L. crispatus* abundance in the vagina during the first trimester of pregnancy^1, 2^ appears to be limited to women with first conceptions. Thus, the contribution of specific vaginal bacteria or groups of bacteria to pregnancy outcome needs to be reevaluated and may differ depending on pregnancy history. Recurrent miscarriages and preeclampsia are most common in nulliparous women^8–10^, indicating that despite having what is generally considered a “healthy” lactobacilli-dominated vaginal microbiota, these women are most susceptible to alterations that interfere with successful pregnancy outcome. *L. crispatus* predominance and lactic acid production are associated with the inhibition of growth of potentially pathogenic bacteria in the vagina^11–13^ and contribute to immune regulation^14–16^ and the maintenance of essential maternal physiological functions^17, 18^. Despite these properties the decrease in *L. crispatus* abundance in women with a prior conception suggest that its influence on pregnancy outcome is reduced in multiparous pregnancies.

The expression of genes that inhibit pro-inflammatory immune system activation are up-regulated during pregnancy to minimize interference with proper fetal and maternal cell differentiation, fetal organ development and pregnancy progression^19^. In addition, extrathymic regulatory FOXP3^+^CD4^+^ T lymphocytes (Treg cells) that recognize paternal antigens expressed by the fetus begin to be produced shortly after implantation of the embryo in the uterus^20–22^. These Treg cells are immunosuppressive and stimulate the release of anti-inflammatory mediators that limit cell-mediated immune responses not only to fetal antigens but also to inflammation induced by bacteria and other antigens that may be encountered by the mother. A population of these Treg cells persist in the circulation after a pregnancy is concluded. Their proliferation is induced by a subsequent conception which results in an elevated level of immunosuppression as compared to that which is present following a first pregnancy^23–25^. Concomitant with variations in Treg-directed immune system modulation in nulliparous and multiparous women the present study identifies an alteration in the composition of the vaginal milieu with parity. Identification of the specific vaginal components that differ between a first and subsequent conception and their contribution to an altered vaginal microbiota and to pregnancy-related immunity remains to be more fully determined.

The association between *L. iners* as the dominant member of the vaginal microbiota and a history of spontaneous abortions is consistent with a previous study showing that this bacterium does not promote maintenance of a stable vaginal microbiota during pregnancy but, instead, is associated with the proliferation of other bacterial genera^14^. The occurrence of a non-lactobacilli-dominated vaginal microbiota during early pregnancy in women with a first conception may increase the likelihood of bacterial migration into the uterus and interfere with immune regulatory mechanisms essential for proper embryo intrauterine implantation. Unfortunately, In the present study we do not have information on whether or not the reported abortions preceded a successful pregnancy in all of our subjects. Further investigations are needed to identify biomarkers that can pinpoint the subset of women with an *L. iners-*dominated vaginal microbiota who are truly at risk for spontaneous abortion.

It should be stressed that the great majority of women in the present study delivered a healthy baby at term following an uneventful gestation, regardless of which bacteria were numerically most abundant in their vaginal microbiota. Thus, while differences in the composition of the vaginal microbiome may influence susceptibility to an adverse pregnancy outcome in a subset of women, these variations do not predispose the majority of women to a pathological event. There clearly remains an unmet need to identify the genetic, environmental, immunological and physiological factors that, when combined with the presence of a specific vaginal microbiota, increase the likelihood of an adverse outcome in individual pregnant women.

Advantages of our study include the relatively high number of subjects evaluated, collection of all samples by a single investigator and utilization of state of the art techniques for analysis of vaginal communities. A limitation is that since all subjects were from a single institution in New York City it remains uncertain if the findings will be similar in women from other geographical locations and racial groups. It is also possible that an unknown number of women in the first conception group had a prior pregnancy that ended in an early stage abortion without the woman’s awareness and that their designation as having no prior exposure to the products of conception is incorrect. Therefore, we cannot rigorously exclude the possibility that this group includes some women with a very early stage pregnancy loss. In addition, it remains unknown whether previous pregnancy history influences the composition of the vaginal microbiota in later pregnancy stages or postpartum. Exposure to the male partner’s semen can elicit immunity to paternal antigens prior to conception^26^. The extent of induction of pre-conception anti-paternal immunity may be expected to vary depending on the length of semen exposure as well as to individual variations in the couple’s genetic, immune and physiological capacity. It would, therefore, be of interest to determine the influence of length of exposure to semen from a specific male partner on the composition of his partner’s vaginal microbiome.

While it appears unlikely to us based on the totality of experimental data, the possibility still remains that differences in unidentified demographic characteristics between the study groups may have contributed, at least in part, to the observed variations in the vaginal microbiota. Further investigation of different study populations should help to more firmly resolve the validity of our data interpretation.

In conclusion, the composition of the vaginal microbiome in women in their first trimester of pregnancy varied depending upon whether they have been previously exposed to the products of conception. This suggests that the influence of specific bacteria or groups of bacteria in the vagina that promote fetal well-being and development in early gestation may differ between primiparous and multiparous women.

## Methods

### Subjects

This prospective study consisted of 155 women who were seen at the outpatient obstetrical service at Weill Cornell Medicine between September 2013 and November 2014 and who were between 8–12 weeks gestation. Selection was based solely on the clinician’s ability to obtain informed consent, collect the requisite samples and transfer them to the laboratory in a timely manner for processing. Each woman’s prior pregnancy history - first conception, prior spontaneous pregnancy loss, previous delivery – was not known at the time of sample collection. This information as well as outcome of the index pregnancy were obtained by chart review only after completion of all laboratory studies. Exclusion criteria from participation were signs or symptoms of a gynecological disorder or infection at the time of examination, multifetal gestation, presence of an endocrine disturbance, steroid or antibiotic treatment in the previous four weeks, vaginal bleeding or the inability to give informed consent. The study was approved by the Institutional Review Board at Weill Cornell Medicine and each participant gave informed written consent. The methods were carried out in accordance with the relevant guidelines and regulations.

### Sample collection

During a routine speculum-based examination secretions and epithelial cells were obtained from the posterior vaginal fornix with cotton swabs. Each swab was vigorously shaken into 1 ml sterile phosphate-buffered saline, centrifuged and the supernatant and epithelial cell pellet fractions immediately aliquoted and stored at −80 °C. An additional sample was collected from the vaginal wall using the Copan Diagnostics ESwab sample collection tubes (Fisher Healthcare, Houston, TX). These samples were immediately frozen at −80 °C until shipped on dry ice to the Forney lab at the University of Idaho for microbiome analysis. All samples utilized in this study were collected from pregnant women by the same investigator (AB).

### Purification of genomic DNA and 16 SrRNA amplicon production

A validated method^27^ was used to extract and purify total genomic DNA from vaginal swabs as described in Supplemental Material. Amplicons of the V-1 to V-3 region of bacterial 16 S rRNA genes in samples were produced by two consecutive rounds of PCR as described in Supplementary Information. The primers used in the first round were those developed by Frank *et al*.^28^ that better maintain the rRNA gene ratio of Lactobacillus to Gardnerella present in the original sample. The second round of PCR attached sample specific barcodes and Illumina sequencing adapters. The concentrations of amplicons were determined using a Pico Green assay (Promega Inc.) and a SpectraMax Gemini XPS fluorometer (Molecular Devices, Sunnyvale, CA), then equal amounts (~100 ng) were pooled in a single tube. The amplicon pool was cleaned to remove short undesirable fragments using the following procedure. First the pool was size selected with using AMPure beads (Beckman Coulter Inc., Pasadena, CA), the product was then run on a 1% gel, excised from the gel, column purified using a Qiagen MinElute PCR purification kit and size selected again with AMPure beads (BeckmanCoulter, Indianapolis, IN). To determine the quality of the amplicons, the pool was PCR amplified with Illumina adaptor specific primers followed by size selection using a DNA1000 chip and an Agilent 2100 Bioanalyzer. The cleaned amplicon pool was then quantified using the KAPA Illumina library quantification kit (KAPA Biosciences) and the Applied Biosystems StepOne plus real-time PCR system. Finally, sequences were obtained using an Illumina MiSeq. 300 paired-end protocol (Illumina, Inc., San Diego, CA) as described in the Supplementary Information.

### Classification of 16 S rRNA gene amplicons

Raw DNA sequence reads from the Illumina MiSeq were assigned to samples and classified as described in Supplementary Material. Reads belonging to the genus Lactobacillus were analyzed further to identify which species of this genus were present in the samples. Blastn was used in BLAST + (Camacho *et al*., 2009) and each read was compared to a database composed of Lactobacillus 16S rRNA gene sequences longer than 1000 bp that were downloaded from NCBI in February 2014). The identity of the different species reported as the best match for each read was recorded, the number of reads assigned to each of the taxa counted and their relative abundance in the different samples calculated. Phylotype rank abundance data were cleaned to include only samples with ≥3,000 reads such that the depth of coverage for each community was sufficient to detect taxa with a relative abundance of at least 0.01 (1%). For constructing phylotype relative abundance tables we used a simple heuristic rule: to be included in a table a phylotype had to either (a) be present in more than one sample at an abundance of 1% or more, or (b) constitute more than 5% of a single community. Phylotypes that did not meet either of these criteria were aggregated into an “Other” category.

### Statistical analyses

Statistical analysis was performed with the GraphPad (Graphpad Software Inc, San Diego, CA) and SPSS v24 (IBM Corp, Armonk, NY) statistical packages. Distribution of categorical variables was compared with the chi-square test or Fisher’s exact test if found with low frequency. Normality of distribution of continuous variables was examined with the Shapiro-Wilk test. Given that all variables were non-normally distributed Kruskall-Wallis and paired Mann-Whitney U tests were used. All p values were 2-sided and the alpha level of statistical significance was set at 0.05.

### Data availability statement

The amplicon sequences will be deposited in the NCBI Short Read Archive (http:/www.ncbi.nlm.nih.gov/sra/).

## Electronic supplementary material

Dataset 1

Dataset 2

Dataset 3

Dataset 4

## References

[1] Romero R (2014). The composition and stability of the vaginal microbiota of normal pregnant women is different from that of non-pregnant women. Microbiome.

[2] MacIntyre DA (2015). The vaginal microbiome during pregnancy and the postpartum period in a European population. Scientific Rep.

[3] Ayre WB (1951). The glycogen-estrogen relationship in the vaginal tract. J Clin Endocrinol Metab.

[4] Spear GT (2014). Human α-amylase present in lower-genital tract mucosal fluid processes glycogen to support vaginal colonization by *Lactobacillus*. J Infect Dis.

[5] DiGiulio DB (2015). temporal and spatial variation of the human microbiota during pregnancy. Proc Natl Acad Sci USA.

[6] Romero R (2014). The vaginal microbiota of pregnant women who subsequently have spontaneous preterm labor and delivery and those with a normal delivery at term. Microbiome.

[7] Heitmann, R.J. *et al*. Maternal T regulatory cell depletion impairs embryo implantation which can be corrected with adoptive T regulatory cell transfer. *Reprod Sci*. pii. 1933719116675054 (2016). 10.1177/1933719116675054 PMC5933084 27834288

[8] Makhlouf MA (2014). Adverse pregnancy outcomes among women with prior spontaneous or induced abortions. Am J Perinatol.

[9] Xiong X, Fraser WD, Demianczuk NN (2002). History of abortion, preterm, term birth, and risk of preeclampsia: a population-based study. Am J Obstet Gynecol.

[10] Borrell A, Stergiotou I (2013). Miscarriage in contemporary maternal-fetal medicine: targeting clinical dilemmas. Ultrasound Obstet Gynecol.

[11] Aldunate M (2015). Antimicrobial and immune modulatory effects of lactic acid and short chain fatty acids produced by vaginal microbiota associated with eubiosis and bacterial vaginosis. Front Physiol.

[12] Linhares IM, Summers PR, Larsen B, Giraldo PC, Witkin SS (2011). Contemporary perspectives on vaginal pH and lactobacilli. Am J Obstet Gynecol.

[13] Witkin SS (2013). Influence of vaginal bacteria and D- and L-lactic acid isomers on vaginal extracellular matrix metalloproteinase inducer: implications for protection against upper genital tract infections. mBio.

[14] Verstraelen H (2009). Longitudinal analysis of the vaginal microflora in pregnancy suggests that *L. crispatus* promotes the stability of the normal vaginal microflora and that *L. gasseri* and *L. iners* are more conducive to the occurrence of abnormal vaginal microflora. BMC Microbiol.

[15] Doerflinger SY, Throop AL, Herbst-Kralovetz MM (2014). Bacteria in the vaginal microbiome alter the innate immune response and barrier properties of the human vaginal epithelia in a species-specific manner. J Infect Dis.

[16] Anahtar MN (2015). Cervicovaginal bacteria are a major modulator of host inflammatory responses in the female genital tract. Immunity.

[17] Kanninen TT, Jayaram A, Jaffe Lifshitz S, Witkin SS (2014). Altered autophagy induction by sera from pregnant women with preeclampsia: a case-control study. BJOG.

[18] Sisti G, Kanninen TT, Witkin SS (2016). Maternal immunity and pregnancy outcome: focus on preconception and autophagy. Genes Immun.

[19] Witkin SS (2011). Unique alterations in infection-induced immune activation during pregnancy. BJOG.

[20] Aluvihare VR, Kallikourdis M, Betz AG (2004). Regulatory T cells mediate maternal tolerance to the fetus. Nat Immunol.

[21] Sasaki Y (2004). Decidual and peripheral blood CD4+CD25+ regulatory T cells in early pregnancy subjects and spontaneous abortion cases. Mol Hum Reprod.

[22] Somerset DA, Zheng Y, Kilby MD, Sansom DM, Drayson MT (2004). Normal human pregnancy is associated with an elevation in the immune suppressive CD25+CD4+ regulatory T-cell subset. Immunology.

[23] Rosenblum, M.D., Way, S.S. & Abbas, A.K. Regulatory T cell memory. Nature Rev Immunol **16**, 90-101, doi:10.1038nri.2015.1 (2016). 10.1038/nri.2015.1 PMC5113825 26688349

[24] Figueiredo AS, Schumacher A (2016). The T helper type17/regulatory T cell paradigm in pregnancy. Immunol.

[25] Williams Z (2012). Inducing tolerance to pregnancy. N Engl J Med.

[26] Robertson SA, Prins JR, Sharkey DJ, Moldenhauer LM (2013). Seminal fluid and the generation of regulatory T cells for embryo implantation. Am J Reprod Immunol.

[27] Yuan S, Cohen DB, Ravel J, Abdo Z, Forney LJ (2012). Evaluation of methods for the extraction and purification of DNA from the human microbiome. PLoS One.

[28] Frank JA (2008). Critical evaluation of two primers commonly used for amplification of bacterial 16S rRNA genes. Appl Environ Microbiol.

