# Supplementary material for: Influence of Pregnancy History on the Vaginal Microbiome of Pregnant Women in their First Trimester
Source: Sci Rep. 2017 Aug 31;7:10201. doi: 10.1038/s41598-017-09857-z (PMC5579028; doi:10.1038/s41598-017-09857-z)
Supplement: Supplementary file 4 — Dataset 4 [file 41598_2017_9857_MOESM4_ESM.doc]

# Supplementary Information

Influence of Pregnancy History on the Vaginal Microbiome of

Pregnant Women in their First Trimester

Dimitrios Nasioudis, Larry J. Forney, G. Maria Schneider, Karol Gliniewicz, Michael France, Allison Boester, Mio Sawai, Jessica Scholl, Steven S. Witkin

## Whole-genomic DNA extraction from vaginal swabs

All archived vaginal swab specimens were thawed on ice and then vortexed for 5 minutes to suspend the cells. A 0.5 ml-aliquot was transferred to a sterile 2.0 mL tube with cell lysis buffer composed of 50 μl lysozyme (10 mg/ml, Sigma-Aldrich), 6 μl mutanolysin (25 KU/ml; Sigma-Aldrich, St. Louis, MO, USA), and 3 μl lysostaphin (4000 U/ml, Sigma-Aldrich) and 41 μl of TE50 buffer (10 mM Tris·HCl and 50 mM EDTA, pH 8.0). After 1 hour of incubation at 37°C, 600 mg of 0.1-mm-diameter zirconia/silica beads (BioSpec, Bartlesville, OK, USA) were added to the mixture and cells were mechanically disrupted using Mini-BeadBeater-96 (BioSpec) at 2100 rpm for 1 minute. Further isolation and purification of the total genomic DNA from crude lysates were processed using QIAamp DNA Mini Kit (Qiagen, Hilden, GER) according to the manufacturer’s recommendation except the DNA was eluted into two separate tubes using two 100μl aliquots of AE buffer (10 mM Tris-HCl, 0.5 mM EDTA; pH 9.0). A PicoGreen assay was used to quantify genomic DNA in each sample (Invitrogen, Carlsbad, CA, USA). Fluorescence was determined using a SynergyTM HT Multi-Mode Microplate Reader (BioTek, Winooski, VT, USA) at an excitation wavelength of 485 nm and emission wavelength of 528 nm.

## PCR amplification and sequencing of the V1-V3 region of bacterial 16S rRNA genes

Amplicons were produced at the University of Idaho using de-identified samples and the work done was exempt from IRB oversight. The variable V1-V3 regions of 16S rRNA genes in each sample were amplified in two rounds of PCR with dual barcode indexing prior to analysis on an Illumina MiSeq platform (Illumina, San Diego, CA, USA). The first PCR round amplified the target specific regions in 16S rRNA genes (E. coli positions 27F-534R), while the second attached sample specific barcodes and Illumina sequencing adapters.

Amplicons of the V1-V3 regions of 16S rRNA genes in samples were made using the 16S rRNA primers 27F and 534R developed by Frank et al. which were shown to better maintain the rRNA gene ratio of *Lactobacillus* to *Gardnerella* present in the original sample. PCR amplification was done in 96-well microtiter plates using AmpliTag Gold DNA polymerase (Applied Biosystems) and 100ng of template DNA in a total reaction volume of 50μl.

The first round of PCR was run in a PTC-100 thermal controller (MJ Research, St. Bruno, Quebec, CAN) using the following cycling parameters: 2 min of denaturation at 95°C, followed by 20 cycles of 1 min at 95°C (denaturing), 1 min at 51 °C (annealing), and 1 min at 72 °C (elongation), with a final extension at 72°C for 10 min. The presence of amplicons was confirmed by agarose gel electrophoresis and staining with SYBRGreen. The second PCR was run in a total reaction volume of 20μl using the following parameters: 10 min of denaturation at 95°C, followed by 10 cycles of 15 s at 95°C (denaturing), 30 s at 51 °C (annealing), and 1 min at 72 °C (elongation), with a final extension at 72 °C for 3 min. Negative controls without a template were included for each primer pair. The concentrations of amplicons were quantified by fluorometry (GeminiXPS, Molecular Devices, Sunnyvale, CA, USA) using PicoGreen, then equimolar amounts (100 ng) of the PCR amplicons were pooled in a single tube. Short DNA fragments and amplification primers were removed from the pool amplicons using AMPure beads (Beckman-Coulter, Indianapolis, IN, USA), and then the purified amplicons were recovered from a 1% agarose gel followed by a second size selection with AMPure beads. The resulting amplicon pool was amplified by PCR using Illumina adaptor specific primers and the PCR product was analyzed on a DNA 1000 chip for the Agilent 2100 Bioanalyzer (Agilent Technologies, Santa Clara, CA, USA). When the entire purification procedure was completed, and no short fragments were observed after PCR, the final amplicon pool was then quantified using the KAPA Illumina library quantification kit (KAPA Biosciences, Wilmington, MA, USA) and the Applied Biosystems StepOne plus real-time PCR system. The de-identified amplicons were sequenced by the Genomics Resources Core of the Institute for Bioinformatics and Evolutionary Studies (IBEST) at the University of Idaho using an Illumina MiSeq platform and a 2 x 250 bp paired-end protocol (Illumina, Inc., San Diego, CA) with custom sequencing primers and 10% phiX DNA to increase sequence diversity.

## Read quality control, sequence analysis and taxonomic assignments

Raw unclipped DNA sequence reads from the Illumina sequencing platform were cleaned, assigned and filtered in the following manner. Raw FASTQ files were sorted based on barcodes in reads 2 and 3 of the Illumina four read protocol, allowing for one mismatch. Amplicon primer sequences in reads 1 and 4 were identified using Cross Match (version 1.080806, parameters: min matches=8, min score=16) from the phred/phrap/consed application suite. Cross Match alignment information was then entered to R and processed to identify alignment quality, directionality, barcode assignment, and read clip points. Base quality clipping was performed using the application Lucy (version 1.20p, parameters: max average error=0.002, max error at ends=0.002). The clipped reads were then aligned to the SILVA bacterial sequence database http://www.arb-silva.de using mothur (www.mothur.org/‎; version 1.27). Alignment end points were identified and used in subsequent filtering. Sequence reads were filtered to only those that met the following criteria: (a) sequences were at least 100 bp in length; (b) max hamming distance of barcode = 1; (c) maximum number of matching error to forward primer sequences = 2; (d) had <2 ambiguous bases (Ns); (e) alignment to the SILVA bacterial database was within 75 bp of the expected alignment start and stop position; and (f) read alignment started within the first 5 bp and extended through read to within the final 5 bp. The RDP Bayesian classifier was used to assign clipped and concatenated (reads 1 and 4) sequences to phylotypes (RDP 2.5; http://rdp.cme.msu.edu). Reads were assigned to the first RDP level with a bootstrap score ≥50. The proportions of various phylotypes in each sample were then calculated.
